# Plant Growth Regulator Residues in Edible Mushrooms: Are They Hazardous?

**DOI:** 10.3390/foods14234098

**Published:** 2025-11-28

**Authors:** Qinghua Yao, Desen Su, Xiuxian Lin, Hui Xu, Yunyun Zheng, Yuwei Xiao

**Affiliations:** 1 Fujian Key Laboratory of Agro-Products Quality and Safety, Institute of Quality Standards Testing Technology for Agro-Products, Fujian Academy of Agricultural Sciences, Fuzhou 350003, China; suds66@163.com (D.S.);; 2 College of Food Science, Fujian Agriculture and Forestry University, Fuzhou 350002, China; xhuifst@163.com (H.X.);

**Keywords:** edible mushroom, plant growth regulator, dietary exposure, risk assessment, probabilistic approach

## Abstract

Mushroom production and economic value on a global scale are significantly increasing. On the other hand, food safety has raised concerns; however, limited research exists on the presence of plant growth regulator (PGR) residues in edible mushrooms. Herein, this study appears to be the first to comprehensively investigate PGR residual characteristics and assess their associated dietary exposure risks to consumers. A total of 105 edible mushroom samples of seven different varieties were analyzed, and the overall detection rate was 81%. The residual level of PGRs ranged from below the limit of detection to 6.308 mg/kg. Among varieties, 100% of *A. aegerita*, *T. fuciformis* Berk, and *H. erinaceus* samples contained at least one PGR residue. Dietary exposure risks were assessed using both deterministic and probabilistic approaches. Calculated values of both %ADI (acceptable daily intake) and %ARfD (acute reference dose)were below 100 and do not indicate a potential health concern with respect to edible mushroom consumption. However, several PGRs had a relatively high %ADI or %ARfD value, suggesting that the Maximum Residual Limits (MRLs) and associated regulatory norms should be immediately established. This work not only provides valuable information for edible mushroom consumers but also an important reference for the risk management decision.

## 1. Introduction

The demand for food, fruits, and vegetables has increased dramatically in recent years due to the continuous growth of the global population [1]. To meet this demand, agricultural production will need to increase by 70% [1]. Among current protein sources, microbial proteins are sustainable, offering high-quality protein at a low cost. Edible mushrooms, the fruiting bodies of macroscopic filamentous fungi, are a valuable choice to partially meet the above demand due to their unique aromatic properties and rich nutrition [2]. They are characterized by high protein content, high essential amino acids, high polysaccharides, low salt, low sugar, and low calories [1,2]. Besides that, mushroom is widely acknowledged for pharmacological activities, such as anti-diabetic, anti-tumor, and anti-viral effects [3,4,5]. Out of 100,000 known species of fungi, more than 2400 species are edible and medicinal mushrooms [1]. Accordingly, mushroom production and economic value have significantly increased on a global scale. Global production of edible mushrooms has increased by about 10% per year and reached 48.336 million tons in 2022 [1,6]. As a result, there has also been an increasing focus on the safety risk associated with hazardous compounds (i.e., pesticides, metals, and mycotoxins) in edible mushrooms.

Plant growth regulators (PGRs), defined as synthetic compounds and natural plant hormones, modulate physiological and biochemical processes in various agricultural practices, including seed germination, breaking dormancy, modulating bud elongation, and inducing flowering and fruiting [7]. Their application plays a vital role in enhancing crop productivity by 10–15 million tons per year [8,9]. PGRs also reduce the dependency on excessive fertilizers and pesticides, thereby promoting sustainable development [7]. These potential benefits of PGR make their market sales significantly increase, with global sales reaching USD 11 billion by 2027 [7,8]. Meanwhile, a few studies demonstrated an inevitable risk to human health due to exposure towards several PGRs via food consumption [10]. Chlormequat chloride is the most commonly used PGR in agriculture and horticulture around the world [11]. It has been recognized that maternal exposure to chlormequat chloride during pregnancy disrupts the normal process of embryo growth and even has adverse effects on postnatal health [12]. In addition to acute poisoning due to the accidental ingestion of PGRs, accumulative poisoning of PGRs can cause disease development and death of livestock and poultry, with severe outcomes such as liver toxicity, neuroinflammatory responses, and decreased autophagy [13,14,15].

As such, ensuring the safety of their residues in agricultural products is crucial. According to the reports of the Ministry of Agriculture of the People’s Republic of China [16] and our previous surveys, PGR residues were widely detected in edible mushrooms. Such compounds may be from their direct application and/or from indirect contamination by substrates (i.e., cottonseed hull and wheat bran) during edible mushroom cultivation. The primary cause may be that PGRs are widely used to improve crop yield in cotton (*Gossypium hirsutum* L.) [17], wheat (*Triticum aestivum* L.) [18], and maize (*Zea mays* L.) [19]. However, of the 209 possible PGRs, only gibberellic acid and triacontanol have been registered for mushrooms in China. And the corresponding MRL was not available for most PGRs [20,21]. Given that, PGR residues can potentially pose significant adverse effects on consumers. Additionally, compared to insecticides [22,23], metals [24,25], and mycotoxins [26,27], there is a lack of comprehensive studies on the investigation of PGR residues in edible mushrooms, and clearly, a risk assessment is required.

Here, focused on 11 PGRs, which may be directly used and/or have potential residues in substrates for edible mushroom cultivation, the present article was designed as follows: (a) An analytical method was validated for the determination of the 11 PGR residues in edible mushrooms; (b) the residual status of PGRs were studied in seven edible mushroom varieties; (c) the dietary risks of the residues were assessed for consumers among different ages, habitations, and sex groups using deterministic and probabilistic approaches. This study could provide data for the safe and reasonable use of PGR-inedible mushrooms and serve as a reference for the establishment of MRLs in China.

## 2. Materials and Methods

### 2.1. Sample Collection and Reagents

A total of 105 individual samples of 7 edible mushroom species, including *Agrocybeaegerita*, *Auricularia auricula*, *Lentinula edodes*, *Pleurotus eryngii*, *Tremella fuciformis* Berk, and *Hericium erinaceus*, were randomly collected from plantations, markets, or wholesalers in accordance with guidelines in China [28]. The collected samples were put in sterile polyethylene bags and immediately transported to the laboratory. And only the edible parts were used for analysis.

Certified standards of paclobutrazol (CAS, 73738-62-0), mepiquat (CAS, 15302-91-7), forchlorfenuron (CAS, 68157-60-8), chlorpropham (CAS, 101-21-3), thidiazuron (CAS, 51707-55-2), diuron (CAS, 330-54-1), diethyl aminoethylhexanoate (CAS, 10369-83-2), flumetralin (CAS, 62924-70-3), pyraflufen-ethyl (CAS, 129630-19-9), pendimethaline (CAS, 40487-42-1), sodium 2-nitrophenoxide (CAS, 824-39-5), sodium 4-nitrophenoxide (CAS, 824-78-2), and sodium 5-nitroguaiacolate (CAS, 67233-85-6) were supplied by Tanmo (Beijing, China). High-performance liquid chromatography (HPLC)-grade acetonitrile, methanol, and formic acid were purchased from Merck (Darmstadt, Germany). Ammonium formate was obtained from Anaqua Chemicals Supply (Cleveland, OH, USA). Sodium chloride (NaCl),anhydrous magnesium sulfate (MgSO_4_) and N-propylethylenediamine (40–60 μm, PSA) were obtained from GL Sciences (Tokyo, Japan).

### 2.2. Samples Pre-Treatment

The PGR analysis and quality control were performed according to our previous research [29]. Briefly, the samples were washed with running water and ground in a blender. A subsample (10 g of a fresh sample or 1 g of a dried sample) was weighed into a 50 mL Teflon centrifuge tube. For dried samples, 9 mL distilled water was added and macerated for 30 min. Then, 10 mL UPLC-grade acetonitrile was added and vortexed immediately for 1 min. A total of 4 g anhydrous MgSO_4_ was added to remove moisture. The tube was sealed and shaken for 1 min. The extracts were centrifuged at 4000 rpm for 5 min. Approximately 2 mL of the supernatant was collected, and 75 mg PSA was added. The mixtures were vigorously shaken for 1 min, followed by incubating for 5 min. Finally, the supernatant was filtered by a 0.22 μm PTFE filter and was then added into auto-sampler vials for further analysis.

### 2.3. LC-MS/MS Analysis

Anultra-fast liquid chromatography system coupled to an 8050 triple quadrupole mass spectrometer (Shimadzu, Kyoto, Japan) was used to detect PGR residues; the ACQUITYUPLCHSST3 (2.1 × 100 mm, 1.8 μm) was applied to the LC system. The mobile phase consisted of eluent A (water containing 2 mmol L^−1^ ammonium formate and 0.01% formic acid) and B (methanol) at a flow rate of 0.3 mL/min. The gradient programs were as follows: initially 2% B; 2 min 2% B; 2.5 min 75% B; 4.9 min 80% B; 8.5 min 100% B; 11.5 min 100% B; 11.6 min 2% B; and 14.0 min, 2% B. The injection volume was 1 μL, and the column temperature was held at 40 °C. Detection of the target compounds was performed by an electrospray ionization source (ESI) in positive and negative modes, and data were acquired in multiple reaction monitoring (MRM) mode. The interface voltage was set to 4.5 kV. Collision-induced dissociation was performed using argon as the collision gas. The interface, de-solvation, and heat block temperatures were set to 300 °C, 526 °C, and 400 °C, respectively. Nitrogen was used as curtain gas. The flow rates of the heating and nebulizer gases were set to 10 L/min and 3 L/min, respectively.

### 2.4. Analytical Method Validation

In line with SANTE guidelines [30], the analytical method was executed using various parameters, including linearity, matrix effects (MEs), repeatability, the limit of detection (LOD), and the limit of quantitation (LOQ). Each recovery experiment was conducted in seven replications. And as per ISO IEC 17025 [31], a quality assurance (QA) program (proficiency testing, spike testing, intra-laboratory testing, and retain and replicate testing) was implemented to ensure the credibility of the data.

### 2.5. Exposure Risk Assessment

Due to being relatively simple, rapid, and inexpensive, deterministic (point estimate) approaches were undertaken first as a screening assessment. The outcome of the exposure assessment is compared to a safe level of intake, such as an *ADI* or an *ARfD*.

The calculation of the chronic (long-term) dietary exposure risk (*%ADI*) assessment was essentially based on four parameters: the residual levels of each PGR (*C*), the average mushroom consumption per day (*F*), the average body weight (*bw*), and the *ADI*. The relevant equation is as follows.

(1) $$\;\%ADI=\frac{C\times F}{bw\times ADI}\times 100$$

The data on edible mushroom consumption and body weights for different subpopulations were acquired from our previous questionnaire-based survey [32].

To assess the acute (short-term) dietary exposure risk (*%ARfD*),

(2) $$\%ARfD=\frac{LP\times HR}{bw\times ARfD}\times 100$$

where *LP* is the large portion (97.5th percentile of eaters, for the general population *LP* = 46.3 g, for children *LP* = 12.7 g, referring to Australia), *HR* is the highest residue in collected samples (mg/kg), and *bw* is body weight (kg).

As listed in Table 1, values of *ADI* or *ARfD* were cited from the Joint FAO/WHO Meeting on Pesticide Residues (JMPR) or the EU pesticide database [33,34].

Non-detects were addressed by the presumption of the best-case scenario lower bound (LB) and were set to zero in the deterministic approaches. In cases where PGR has a relatively higher risk, probabilistic (stochastic) models were then used to provide more details on the distribution of exposed consumers. Non-detects were substituted by LOD to guarantee the result would be pessimistic. And a *%ADI* or *%ARfD* of less than 100 would be considered as an acceptable risk to health; otherwise, there is an unacceptable risk. A higher value indicates a greater exposure risk.

### 2.6. Statistical Analysis

Data analysis was performed using Microsoft Office Excel 2007 and SPSS Statistics 26 for Windows (SPSS Inc., Chicago, IL, USA). Mean residual levels of each detected PGR in different varieties were evaluated by one-way analysis of variance (ANOVA). A value of *p* < 0.05 was considered statistically significant.

## 3. Results and Discussion

### 3.1. Method Validation

The analytical method has been validated and meets the SANTE/11312/2021 standard. The detailed results are summarized in the Supplementary Materials. The LOD and LOQ were considered within the lowest concentration, achieving a signal-to-noise ratio (S/N) of 3, and the lowest spiked level met a satisfactory accuracy and precision. The obtained LODs and LOQs were 0.1–5.0 μg/kg and 0.4–16.5 μg/kg, respectively. Excellent linearities were obtained in the range of 0.0004–10 μg/kg with a value of R^2^ higher than 0.999. Extraction efficiency in terms of recovery was determined at low, medium, and high spiked levels. The percentage of recovery ranged from 69.7 to 103.1% and from 71.2 to 103.3%, respectively, for intra-day and inter-day. An RSD of 0.5–9.0% indicated good precision of the methodology. The matrix effect (ME) was calculated by the slope ratio of the matrix-matched calibration curve and the solvent standards calibration curve.ME could be ignored when the value was within 0.8–1.2; otherwise, it was regarded as the matrix suppression or enhancement effect [35]. In the current study, the ME values indicated that matrix suppression existed in two analytes (diethyl aminoethylhexanoate and thidiazuron), while matrix enhancement existed in one analyte (sodium 4-nitrophenoxide). Hence, matrix-matched calibration was applied for quantitative analysis. Based on these results, the proposed method could be applied to detect the targeted PGR residues in edible mushrooms. The detailed data for method validation could be found in Tables S1–S3.

### 3.2. PGR Residues in Edible Mushrooms

**Figure 1:**
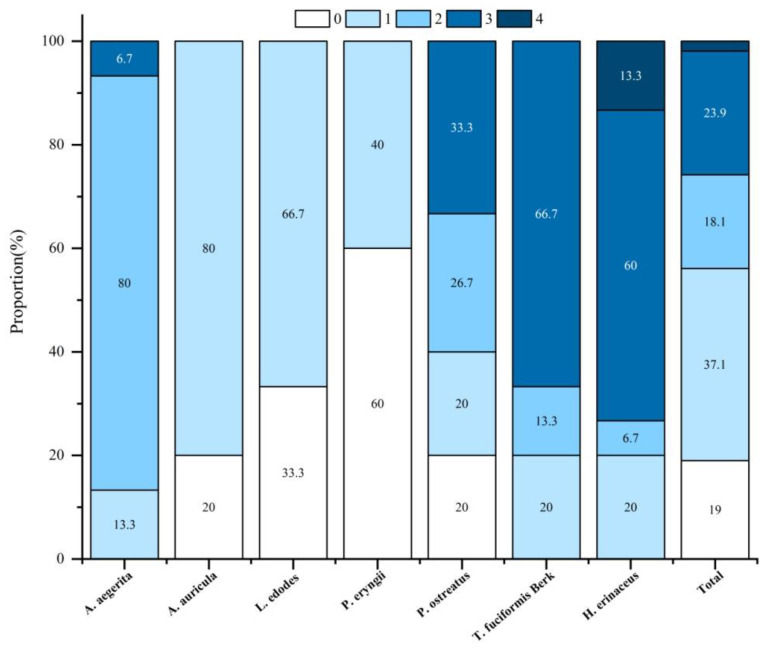
The occurrence of pesticide residues in edible mushrooms.

**Figure 2:**
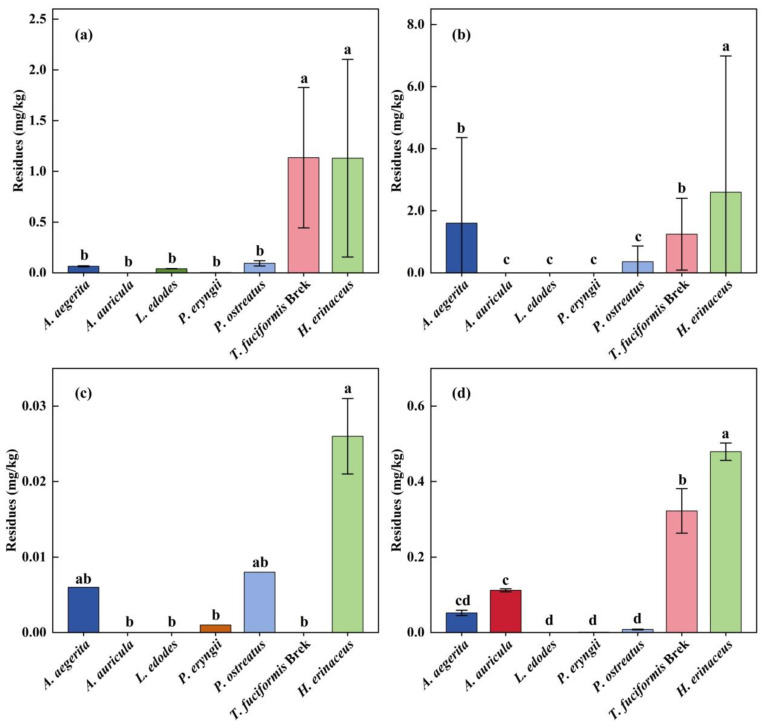
Residual levels of PGRs in edible mushrooms. (**a**) Chlormequat; (**b**) mepiquat chloride; (**c**) thidiazuron; and (**d**) sodium nitrophenolate. ^a,b,c,d^ Different letters indicate significant differences (*p* < 0.05).

The presence of PGR in edible mushrooms may not be from their direct application but mainly from sterilized substrates. Table 1 shows the information on the 11 PGRs, including the mode of action (MOA), ADI, ARfD, and MRLs. Among the 105 samples tested, only 19.0% of the analyzed samples were found below LOD. Chlormequat was the most detected, with a detection frequency of 52.4%, followed by mepiquat chloride (44.8%), sodium nitrophenolate (47.6%), and thidiazuron (7.6%), and the average residual level was 0.352, 0.829, 0.139, and 0.006 mg/kg. These four PGRs are not authorized in China for edible mushrooms, so they resulted from unregulated use. Concerning the different varieties investigated, PGR residues were detected most often in *A. aegerita*, *T. fuciformis* Berk, and *H. erinaceus*, in which 100% of the samples above LOD (Figure 1). Figure 2 shows the residual levels of positive PGRs in seven edible mushroom varieties. On average, the highest concentrations of mepiquat chloride, thidiazuron, and sodium nitrophenolate were all determined in *H. erinaceus*. For chlormequat, its residual level in *T. fuciformis* Berk or *H. erinaceus* is significantly higher than that of five other varieties (*p* < 0.05). Obviously, considering the detection rates and residual levels, PGR residues in *T. fuciformis* Berk, *H. erinaceus,* and *A. aegerita* should be a crucial concern. The detected PGR and residual levels in different varieties are listed in Table 2. Mepiquat chloride in *A. aegerita*, *T. fuciformis* Berk, and *H. erinaceus*; sodium nitrophenolate in *A. auricula*; and chlormequat in *T.fuciformis* Berk and *H. erinaceus* were detected, with a detection rate higher than 80%. This indicated that the PGRs were probably misused by mushroom farmers due to the lack of registration. The other potential source was that PGRs already existed in sterilized substrates such as seed hulls and wheat bran and might be absorbed by mushrooms [36]. Thus, PGR residues were found in almost all *T. fuciformis* Berk, *H. erinaceus*, and *A. aegerita* samples. Furthermore, multi-residues cannot be ignored due to their combined toxicity [37]. In cotton, wheat, or corn cultivation, to achieve the desired regulation effects, various PGRs may be simultaneously used. The phenomenon of PGR multi-residues had been commonly observed in cottonseed hull [29]. As shown in Figure 1, the multi-residues were detected in 43.9% of whole 105 samples, and up to four different pesticides were detected in individual samples. Among different varieties, the following trend was shown: *A. aegerita* (86.7%), *T. fuciformis* Berk (80%), *H. erinaceus* (80%), and *P. ostreatus* (60%). For *A. auricula*, *L. edodes*, and *P. eryngii,* multiple residues had not been detected. Notably, the MRLs of 11 targeted PGRs have not been established for edible mushrooms in China [20,21]; it is herein essential to assess the risks of dietary exposure to these PGR residues.

### 3.3. Deterministic Intake Calculations

#### 3.3.1. Long-Term Intake and Chronic Exposure Risk

**Figure 3:**
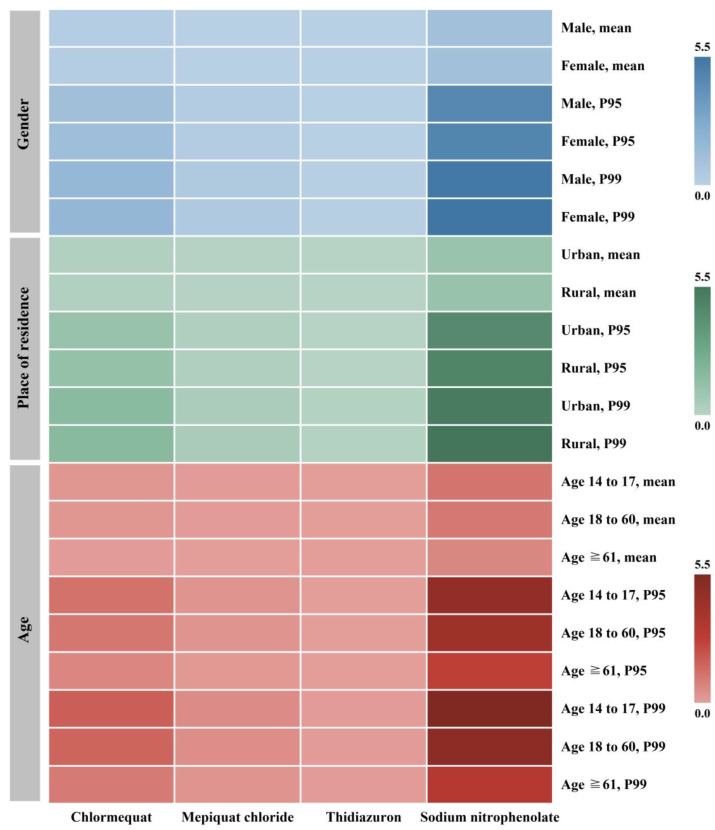
Chronic dietary exposure risk of PGR residues in edible mushrooms for different subpopulations.

The dietary exposure to detected PGRs has been calculated for different subpopulations under the best-case scenario using different distribution models, and the results are shown in Figure 3. Thidiazuron and sodium nitrophenolate were recorded minimum and maximum exposure, respectively. The relatively higher risks from sodium nitrophenolate are attributed to its extremely low value of ADI (0.003 mg/kg bw/day). For the general population, the exposure risk values varied from 0.002 to 1.098 at the mean value distribution model, and from 0.060 to 5.103 when using the 99th percentile of residue-level distribution. These indicated that the chronic risk from PGR exposure via edible mushroom intake could be acceptable. Considering the gender, age, and habitation of consumers, females, adults aged 18 to 60, and rural residents had elevated exposure risks compared to their corresponding counterparts. This might be due to their lower body weight or higher daily food intake. The present results were similar to studies of some other undesirable risk factors in diverse foods, such as perchlorate in vegetables [38], mycotoxins in coix seed [39], and organochlorine pesticide residues in seaweed [40]. In addition, it is also worth noting that there are some susceptible populations such as pregnant women, infants, and young children due to their particular dietary patterns or immature defense systems [41,42,43].

#### 3.3.2. Short-Term Intake and Acute Exposure Risk

According to FAO recommendations, an acute dietary exposure risk assessment needs to be conducted for foods that are subject to potential hazards after a brief period of consumption [44].In this study, the acute dietary exposure risk of chlormequat and mepiquat chloride was assessed for the general population and children, excluding thidiazuron and sodium nitrophenolate due to the lack of *ARfD*. For the general population, *%ARfD* values of chlormequat and mepiquat chloride were 2.73 and 0.79, respectively; the corresponding values were 2.85 and 0.83 for children. Both were significantly below 100 and lower than the *%ARfD* of some pesticides such as endosulfan [23]. That is, acute dietary exposure risk towards PGR residues via consumption of edible mushrooms was considered acceptable. Meanwhile, the aforementioned result simplied that, as a special group, children had a slightly higher potential risk compared to the general population.

### 3.4. Probabilistic Intake Calculation

Due to the obvious limitations of being unrealistic in deterministic intake calculations and less informative in PGR residues in other food sources (i.e., vegetables, fruits, and wheat flour), the probabilistic assessment was herein performed with the Microsoft Office Excel add-on Crystal Ball software, based on the Monte Carlo simulation, to reduce uncertainties. This technique involves the random sampling of each probability distribution to produce thousands or even hundreds of thousands of iterations [45]. In our study, the number of iterations was set to 10,000.

**Figure 4:**
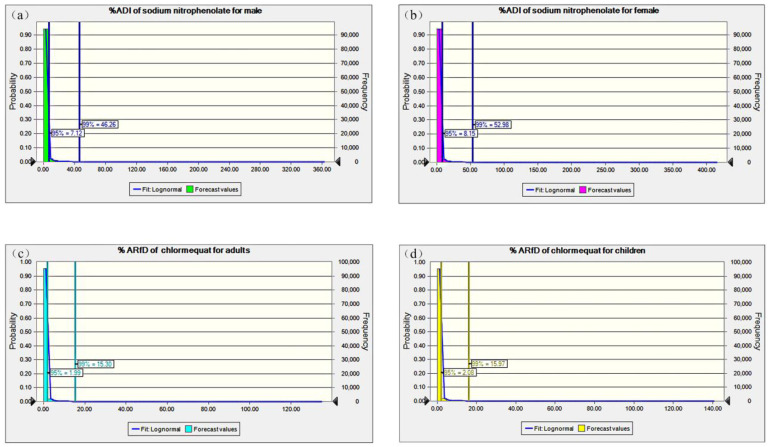
Monte Carlo simulation of the dietary exposure to sodium nitrophenolate and chlormequat residues in edible mushrooms. (**a**) %ADI of sodium nitrophenolate for males; (**b**) %ADI of sodium nitrophenolate for females; (**c**) %ARfD of chlormequat for adults; and (**d**) %ARfD of chlormequat for children.

Consequently, the 95th and 99th percentiles of the dietary exposure risk distribution (%ADI of sodium nitrophenolate and %ARfD of chlormequat), respectively, based on the Monte Carlo simulation, were described for different subpopulations, as shown in Figure 4. The variable of the chlormequat residual level in edible mushrooms had a lognormal distribution. %ADIs with 95th and 99th certainty for sodium nitrophenolate in males were equal to 7.12 and 46.26, respectively, corresponding to 8.15 and 52.98 for females; %ARfDs with 95th and 99th certainty were obtained as 1.99 and 15.30 for adults and 2.08 and 15.97 for children. The results once again advised that PGR residues in edible mushrooms did not pose an unacceptable health hazard but were still the focus of monitoring.

### 3.5. Strengths, Limitations, and Future Perspectives

On the whole, we provide scientific data on the occurrence of PGRs in edible mushrooms and the associated dietary exposure risks for different consumers for the first time. However, the limitations should be clearly addressed to enhance the strength of risk assessment results. For example, although most edible mushrooms are consumed during various culinary processes, processing factors have not been considered in this practice. A few studies have indicated that common household processes such as washing, soaking, blanching, and cooking have been demonstrated to play a role in the reduction in pesticide residues [46,47]. For instance, the average processing factor of detergent solution soaking for pesticide residues in cowpea was 0.22, while it was 0.47 for water soaking [46]. Moreover, for systemic and permeable pesticides (i.e., dinotefuran, dimethoate, and indoxacarb), the removal was lower than 50% in these processing steps [46]. Additionally, it is known that ingested food cannot be directly absorbed and needs to be digested in the gastrointestinal tract [48,49]. The bioactivity of a compound depends on the amount that actually reaches the site of action within the human body. Especially for hydrophobic substances, the overall bioavailability is strongly dependent on their bioaccessibility [46]. A study conducted by Wang et al. revealed that only a small percentage of pesticide residues in fish may enter the circulatory system after ingestion [50]. There is also some evidence that the composition and structure of the food matrix have an impact on bioaccessibility [46]. Hence, it may have overstated health concerns, since not all PGR residues in edible mushrooms are bioavailable. Furthermore, no reliable data existed on the consumption of large portions of edible mushrooms in China. Thus, the acute dietary exposure risk was calculated using the consumption data in other countries. For chronic dietary exposure risk calculation, although the consumption data were derived from our previous survey, several uncertainties still existed due no food consumption survey methodology was able to reflect the true food intake [51]. Finally, it should be kept in mind that the sampling sizes are limited in this study, but they give a general view of the actual situation of PGR residues in edible mushrooms. Generally, all these items are important and effective factors for future studies to do a thorough risk assessment.

## 4. Conclusions

In this paper, a reliable analytical method was developed to determine PGR residues in edible mushrooms, and then a comprehensive survey and risk assessment were conducted using both deterministic and probabilistic models. The results showed that there were widespread PGR residues in edible mushrooms. Among the targeted analytes, four PGRs, including mepiquat chloride, thidiazuron, and sodium nitrophenolate, were detected, with the detection rate of chlormequat ranking first. Notably, 100% of *A. aegerita, T. fuciformis* Berk, and *H. erinaceus* samples contained PGR residues. In terms of the multiple residues, the combination of two or more PGRs was frequent in edible mushrooms, with a detection rate of 43.9%. According to the results of dietary exposure assessments, although both the chronic and acute risks were acceptable, it should warrant concern that the risk indices of several PGRs were already relatively high, even when only considering edible mushroom consumption. Thus, corresponding MRLs and regulatory norms should be immediately established to protect consumer health. And it is necessary to further investigate the actual source of PGR residues in edible mushrooms. Findings will assist stakeholders in effectively managing PGR applications.

## Figures and Tables

**Table 1:** Mode of action (MOA), ADI, ARfD, and MRLs of PGRs.

PGRs	MOA	ADI (mg/kg bw/Day)	ARfD (mg/kg bw)	Registered for Cotton	Registered for Wheat	Registered for Corn	Source
Paclobutrazo	inhibiting gibberellin synthesis	0.022	0.1	N	Y	N	11/55/EU
Uniconazol	inhibiting gibberellin synthesis	0.02	N.A.	Y	Y	N	GB2763
Chlormequa	inhibiting gibberellin synthesis	0.04	0.09	Y	Y	Y	EFSA08
Mepiquat chloride	inhibiting gibberellin synthesis	0.3	0.6	Y	Y	Y	JMPR2023
Thidiazuron	mimicking the effects of both auxin and cytokinin	0.04	N.A.	Y	Y	Y	GB2763
Diethyl aminoethyl hexanoate	stimulating the synthesis of chlorophyll, protein, and nucleic acid	0.023	N.A.	Y	N	Y	GB2763
Sodium nitrophenolate *	enhancing cell viability and facilitating cellular protoplasmic flow	0.003	N.A.	N	Y	N	GB2763
Flumetrali	inhibitors of stem elongation and branching	0.015	0.1	Y	N	N	2015/2105/EU
Diuron	disrupting plastoquinone-mediated electron transfer	0.007	0.016	Y	N	N	Dir 08/91
Pyraflufen-ethyl	inhibiting protoporphyrinogen oxidase	0.2	0.2	Y	Y	N	2016/182/EU
Pendimethalin	inhibiting mitosis	0.1	1	Y	N	Y	JMPR2016

* Containing three compounds: sodium 2-nitrophenoxide, sodium 4-nitrophenoxide, and sodium 5-Nitroguaiacolate. Y, yes. N, no.

**Table 2:** The levels and detection frequency of pesticide residues in edible mushrooms.

Mushroom (No. of Samples)	Pesticides	N > LOQ	Residual Levels (mg/kg)		
			Range	Mean	Median
*A. aegerita* (15)	Chlormequat	8 (53.3%)	<LOD − 0.206	0.064	0.061
	Mepiquat chloride	14 (93.3%)	<LOD − 4.611	1.602	0.351
	Thidiazuron	2 (13.3%)	<LOD − 0.054	0.006	0.000
	Sodium nitrophenolate	5 (33.3%)	<LOD − 0.267	0.052	0.000
*A. auricula* (15)	Sodium nitrophenolate	12 (80.0%)	<LOD − 0.207	0.112	0.115
*L. edodes* (15)	Chlormequat	10 (66.7%)	<LOD − 0.074	0.041	0.054
*P. eryngii* (15)	Chlormequat	1 (6.7%)	<LOD − 0.044	0.003	0.000
	Thidiazuron	1 (6.7%)	<LOD − 0.020	0.001	0.000
	Sodium nitrophenolate	4 (26.7%)	<LOD − 0.008	0.001	0.000
*P. ostreatus* (15)	Chlormequat	9 (60.0%)	<LOD − 0.633	0.094	0.007
	Mepiquat chloride	9 (60.0%)	<LOD − 2.466	0.358	0.059
	Thidiazuron	3 (20.0%)	<LOD − 0.050	0.008	0.000
	Sodium nitrophenolate	5 (33.3%)	<LOD − 0.093	0.008	0.000
*T. fuciformis* Berk (15)	Chlormequat	15 (100%)	0.156–2.812-	1.135	1.261
	Mepiquat chloride	12 (80.0%)	<LOD − 3.022	1.246	1.322
	Sodium nitrophenolate	10 (66.7%)	<LOD − 0.687	0.322	0.378
*H. erinaceus* (15)	Chlormequat	12 (80.0%)	<LOD − 3.259	1.130	0.897
	Mepiquat chloride	12 (80.0%)	<LOD − 6.308	2.600	3.113
	Thidiazuron	2 (13.3%)	<LOD − 0.212	0.026	0.000
	Sodium nitrophenolate	14 (93.3%)	<LOD − 0.647	0.479	0.502

## Data Availability

The original contributions presented in this study are included in the article and supplementary material. Further inquiries can be directed to the corresponding author.

## Supplementary Materials

The following supporting information can be downloaded at: https://www.mdpi.com/article/10.3390/foods14234098/s1, Table S1. CAS registry number and mass spectrometric parameters of 15 PGRs. Table S2. Retention times, MRM conditions, linearity ranges, calibration curve information, LODs, and LOQs for PGRs. Table S3. Recovery, intra-day, and inter-day precisions for PGRs.
