# Supplementary material for: Plant Growth Regulator Residues in Edible Mushrooms: Are They Hazardous?
Source: Foods. 2025 Nov 28;14(23):4098. doi: 10.3390/foods14234098 (PMC12691735; doi:10.3390/foods14234098)
Supplement: Supplementary file 1 [file foods-14-04098-s001.zip › Supplementary data.pdf]

Supplementary Material

Plant growth regulator residues in edible mushrooms. Is it hazardous?

Qinghua Yao [a*], Desen Su [a], Xiuxiang Lin [a], Hui Xu [b], Yunyun Zheng [a], Yuwei Xiao [b]

[a]*Institute of Quality Standards Testing Technology for Agro-products,Fujian Key Laboratory of Agro-Products Quality and Safety, Fujian Academy of Agricultural Sciences,Fuzhou, 350003, China*

[b]*College of Food Science, Fujian Agriculture and Forestry University, Fuzhou, 350002, China*

***Corresponding Author:** Dr. Qinghua Yao

Tel.: +86-591-87869472

Fax: +86-591-87869422;

E-mail: yaoqh24@163.com

Table S legends

Table S1. CAS registry number and mass spectrometric parameters of 15 PGRs.
Table S2. Retention times, MRM conditions, linearity ranges, calibration curve information, LODs, and LOQs for PGRs
Table S3. Recovery, intra-day, inter-day precisions for PGRs.

Table S1 CAS registry number and mass spectrometric parameters of 15 PGRs.

Analyte	CAS	ESI	Adduction	Quantitative				Confirmation			
				Ion pairs (*m/z*)	Q1 Pre Bias/ V)	Collision energy (eV)	Q3 Pre Bias/ V	Ion pairs (*m/z*)	Q1 Pre Bias/ V)	Collision energy (eV)	Q3 Pre Bias/ V
Paclobutrazol	76738-62-0	ESI+	$[M+H]^+$	294.1>70.1	-15	-21	-28	294.1>125.1	-15	-40	-22
Uniconazole	83657-22-1	ESI+	$[M+H]^+$	292.1>70.1	-21	-24	-27	292.1>125.0	-21	-28	-23
Chlormequat	999-81-5	ESI+	$[M+H]^+$	122.1>58.1	-30	-29	-23	122.1>63.0	-30	-22	-24
Mepiquat chloride	15302-91-7	ESI+	$[M+H]^+$	114.2>98.2	-12	-28	-19	114.2>58.2	-12	-27	-22
Thidiazuron	51707-55-2	ESI+	$[M+H]^+$	221.2>102.0	-15	-16	-18	221.2>128.0	-15	-17	-23
Diuron	330-54-1	ESI+	$[M+H]^+$	233.0>72.0	-26	-21	-27	233.0>160.1	-26	-26	-29
Diethyl aminoethyl hexanoate	10369-83-2	ESI+	$[M+H]^+$	216.2>143.2	-20	-16	-20	216.2>100.2	-14	-16	-21
Flumetralin	62924-70-3	ESI+	$[M+H]^+$	422.1>143.1	-11	-20	-29	422.1>107.0	-11	-55	-21
Pyraflufen-ethyl	129630-19-9	ESI+	$[M+H]^+$	413.0>339.0	-11	-20	-24	413.0>253.1	-11	-35	-18
Pendimethaline	40487-42-1	ESI+	$[M+H]^+$	282.2>212.2	-13	-12	-15	282.2>194.1	-10	-20	-21
Sodium 2-nitrophenoxide	824-39-5	ESI-	$[M-H]^-$	138.1>108.0	14	18	23	138.1>91.9	27	22	15
Sodium 4-nitrophenoxide	824-78-2	ESI-	$[M-H]^-$	138.1>108.0	15	19	22	138.1>91.9	15	24	19
Sodium 5-nitroguaiacolate	67233-85-6	ESI-	$[M-H]^-$	168.2>153.1	30	14	14	168.2>123.2	17	19	22

Table S2. Retention times, MRM conditions, linearity ranges, calibration curve information, LODs, and LOQs for PGRs

Analyte	Retention time (min)	ME/%	Equation	R^2	Linear Range ng/mL	LOD μg/kg	LOQ μg/kg
Paclobutrazol	6.38	85.0	y=65517.3x-248484.8	0.9991	0.80~825	0.1	0.4
Uniconazole	6.81	86.7	y=35577.9x-18594.4	1.0000	0.80~415	0.1	0.4
Chlormequat	1.04	86.3	y=14732.9x+2422.4	0.9996	1.25~125	0.1	0.4
Mepiquat	1.09	98.0	y=19230.3x+345153.0	0.9993	2.50~2500	0.5	1.7
Thidiazuron	5.67	71.5	y=16119.6x-53214.4	0.9994	0.80~825	0.1	0.4
Diuron	6.07	89.7	y=48471.3x-76398.4	0.9994	0.40~425	2.0	6.6
Diethyl aminoethyl hexanoate	5.34	45.4	y=104144.6x-442701.6	0.9996	0.80~825	0.3	1.0
Flumetralin	9.11	83.1	y=3706.4x-139746.9	0.9991	8.00~4000	2.0	6.6
Pyraflufen-ethyl	7.02	92.3	y=148248.1x-238003.0	0.9996	0.40~425	0.1	0.4
Pendimethaline	8.95	91.8	y=50813.5x-34590.6	0.9997	0.40~215	0.1	0.4
Sodium 2-nitrophenoxide	5.73	89.7	y=88.6x+9655.3	0.9992	20.00~10000	5.0	16.5
Sodium 4-nitrophenoxide	5.49	95.3	y=7514.3x+163631.7	0.9995	0.40~425	0.1	0.4
Sodium 5-nitroguaiacolate	5.45	118	y=2713.2x-21453.5	0.9999	4.00~4000	0.1	0.4

Table S3. Recovery, intra-day, inter-day precisions for PGRs.

Analyte	Spike levels ng/mL	Recovery (%)		RSD (%)	
		Intra-day (n=7)	Inter-day (3 d)	Intra-day (n=7)	Inter-day (3 d)
Paclobutrazol	20	103.1	103.3	3.1	3.7
	80	99.3	99.1	1.0	4.1
	200	92.3	93.3	0.5	3.3
Uniconazole	20	91.3	90.6	4.4	6.1

	80	102	102	0.6	5.4
	200	101	101	1.3	5.0
Chlormequat	30	73.7	78.3	6.8	5.8
	120	80.5	84.5	1.3	4.9
	300	77.9	83.2	2.4	6.4
Mepiquat	60	71.4	79.2	9.8	9.9
	250	82.0	86.3	2.8	5.1
	600	87.6	94.3	1.0	6.5
Thidiazuron	20	90.5	92.6	3.7	2.0
	80	89.1	91.1	1.8	2.0
	200	84.6	86.9	1.5	2.6
Diuron	10	88.8	91.4	2.8	2.9
	40	88.5	90.9	0.5	2.5
	100	84.4	87.7	1.5	3.5
Diethyl aminoethyl hexanoate	20	105	108	0.6	2.7
	80	102	102	1.4	0.5
	200	90.7	91.1	2.6	1.6
Flumetralin	200	84.9	89.7	4.0	4.7
	800	82.3	86.6	2.3	4.5
	2000	79.4	84.6	1.2	5.4
Pyraflufen-ethyl	10	93.0	95.5	4.2	2.8
	40	93.8	96.7	1.8	3.0
	100	91.5	95.9	1.2	4.1
Pendimethaline	10	84.8	86.1	3.5	1.7
	40	96.2	98.4	1.2	2.0
	100	93.9	95.6	0.8	1.6

	500	69.7	71.2	8.8	3.1
Sodium 2-nitrophenoxide	2000	93.9	94.5	6.9	6.8
	5000	94.2	94.6	4.5	4.7
	10	85.2	91.7	9.0	7.4
Sodium 4-nitrophenoxide	40	75.1	81.2	6.1	7.1
	100	73.4	78.6	1.6	6.6
	100	86.1	89.9	5.9	4.4
Sodium 5-nitroguaiacolate	400	93.3	97.3	2.2	5.2
	1000	90.2	93.7	1.6	4.8
